# Complex-Valued Phase Transmittance RBF Neural Networks for Massive MIMO-OFDM Receivers

**DOI:** 10.3390/s21248200

**Published:** 2021-12-08

**Authors:** Jonathan Aguiar Soares, Kayol Soares Mayer, Fernando César Comparsi de Castro, Dalton Soares Arantes

**Affiliations:** 1 Department of Communications, School of Electrical and Computer Engineering, University of Campinas, Campinas 13083-852, Brazil; kayol@decom.fee.unicamp.br (K.S.M.); dalton@unicamp.br (D.S.A.); 2 Department of Electronics and Computing, Technology Center, Federal University of Santa Maria, Santa Maria 97105-900, Brazil; fernando.castro@ufsm.br

**Keywords:** artificial neural networks, phase transmittance radial basis function, massive MIMO, MIMO decoding, 5G

## Abstract

Multi-input multi-output (MIMO) transmission schemes have become the techniques of choice for increasing spectral efficiency in bandwidth-congested areas. However, the design of cost-effective receivers for MIMO channels remains a challenging task. The maximum likelihood detector can achieve excellent performance—usually, the best performance—but its computational complexity is a limiting factor in practical implementation. In the present work, a novel MIMO scheme using a practically feasible decoding algorithm based on the phase transmittance radial basis function (PTRBF) neural network is proposed. For some practical scenarios, the proposed scheme achieves improved receiver performance with lower computational complexity relative to the maximum likelihood decoding, thus substantially increasing the applicability of the algorithm. Simulation results are presented for MIMO-OFDM under 5G wireless Rayleigh channels so that a fair performance comparison with other reference techniques can be established.

## 1. Introduction

In recent years, with the increasing demand for the real-time processing of big data, the Internet of Things (IoT), and 4K video streaming, technologies to increase area throughput [1] in base station (BS) coverage and hotspot tiers [2] have become increasingly important. In general, the system throughput can be improved by three independent factors: the number of BSs, bandwidth, and spectral efficiency. While the number of base stations is a complicated variable to handle, there are substantial bandwidths in the millimeter wavelength (mmWave) bands that could be employed for BS hotspot tiers. On the other hand, as objects and human bodies easily block mmWaves, increasing the spectral efficiency (SE) of BS coverage tiers arises as a potential solution for wide-area coverage. In order to increase SE, advanced techniques are necessary to use the available BSs and bandwidth more efficiently. In view of this, both BSs and user equipment (UE) currently operate with multiple antennas and orthogonal frequency-division multiplexing (OFDM) [3,4,5] to increase spectral efficiency.

Multicarrier modulation schemes, such as OFDM, have been widely employed in digital communications systems due to their low susceptibility to intersymbol interference (ISI) [6,7,8,9]. OFDM divides the channel bandwidth into *K* orthogonal subcarriers [10]. The serial stream at a high data rate applied to the OFDM input is first converted to multiple parallel low transmission rate sub-streams. Each of the *K* parallel sub-streams modulates one of the *K* subcarriers. In this way, the OFDM symbol duration is *K* times longer than the symbol duration of the equivalent single carrier system, thus avoiding ISI [11]. Another important characteristic of OFDM systems is that multiple users can be multiplexed in frequency, using *K* subcarriers.

Multiple-input and multiple-output (MIMO) technologies use multiple antennas on the transmitter and receiver sides, increasing the wireless channel capacity without extra bandwidth, extra power transmission, or both [11]. Usually, as BSs have more computational power than UEs, a larger number of antennas is applied at the transmitter. In this context, when the number of antennas exceeds the number of users, the term massive MIMO (mMIMO) is frequently used. Generally, mMIMO systems operate with 16 or more antennas in BSs. In addition, the uplink can be composed of one or more UEs, where the former characterizes a single-user mMIMO (SU-mMIMO) [12] and the latter a multi-user mMIMO (MU-mMIMO) [13]. For 6G technologies, ultra-massive MIMO (UM-MIMO) schemes have been explored to support data throughputs of Terabits [14]. MIMO and mMIMO communications are broadly implemented either by beamforming, space–time block codes (STBC), or both [15,16]. While beamforming techniques (either analog, digital, or hybrid [17]) may be more attractive to BSs, because of the necessity of many transmitting antennas, STBC, on the other hand, is feasible for use in both downlink and uplink connections.

The combination of OFDM and MIMO is a major trend in mobile communication systems [18,19,20,21,22], such as 5G and next-generations, which are based on the so-called MIMO-OFDM and mMIMO-OFDM approaches [23]. Nonetheless, digital communication systems over wireless channels may suffer severe signal degradations due to multipath propagation, additive white Gaussian noise (AWGN) [24,25], and Doppler effects [26]. Moreover, another frequent impairment in OFDM systems is signal distortion, characterized by the PAPR (peak-to-average power ratio), due to nonlinearities at the high-power transmitter amplifier [27,28]. Since nonlinear impairments usually degrade the performance of linear filters, the search for robust nonlinear filters at the receiver is essential to circumvent this issue [11,27,29].

Along with the application of nonlinear filters designed for specific problems in telecommunications, artificial neural networks (ANNs) have been extensively studied in various challenging areas of digital communications, including soft and hard fault detection, channel estimation, equalization, and beamforming [30,31,32,33,34,35,36,37,38,39,40]. Neural networks can operate like nonlinear filters, in a structure that can be modeled by nonlinear activation functions, as in multilayer perceptrons (MLPs), or by Gaussian neurons in radial basis function neural networks (RBFNN) [35]. RBFNN Gaussian neurons have two free parameters, namely the Gaussian centers and the variances. Moreover, there is a linear free parameter vector of weights, which linearly weighs the neuron outputs to yield the network output [35,41]. With these three independent sets of parameters, RBFNNs are able to represent high-order nonlinear surfaces without increasing the number of layers, thereby reducing complexity compared with deep neural networks [11,35].

In this context, this work proposes a novel complex-valued RBF neural network architecture: a multiple-input multiple-output phase transmittance RBF (MIMO-PTRBF) neural network for channel estimation and symbol detection in massive MIMO-OFDM communication systems. The PTRBF neural network model was chosen due to its lower computational complexity when compared with deep neural networks and due to its crucial role in avoiding the phase invariance which occurs in a standard complex-valued RBFNN [11,35,41]. The proposed MIMO-PTRBF is an extension to multiple outputs of the single-output PTRBF neural network presented in [41]. This work is based on [35], in which the authors redesigned the PTRBF neural network of [41] to obtain a low-complexity MIMO beamforming transmitter with the complex-MIMO RBF (CMM-RBF). In the present paper, using the Gaussian neuron output bounds presented in [35], we prove convergence in the mean of the PTRBF neural network, relaxing the condition of the trans-dimensional transformation of the complex-valued Gaussian neuron layer. Preliminary results indicate that the proposed architecture competes quite favorably with the conventional MIMO quasi-orthogonal space–time block coding (QOSTBC) with maximum likelihood (ML) decoding based on linear processing [11]. In addition, an STBC coding algorithm with full-rate and half-diversity was developed for massive MIMO coding with square matrices. However, because of its high computational complexity, the ML decoding may be unfeasible for mMIMO with a large number of antennas, as opposed to the MIMO-PTRBF neural network proposed here. Although other decoding techniques with lower computational complexities could be taken into account, for performance comparison, we chose ML decoding because of its optimal performance in linear channels. For example, the sphere decoding has lower computational complexity compared to the ML decoding, but its performance is only lower-bounded by the ML decoding [42]. Furthermore, the MIMO-PTRBF is able to both estimate the channel and decode the received signal, relying on a training sequence. Simulation results show that the proposed architecture achieves significantly improved BER figures when compared with MIMO QOSTBC in an equal scenario, either with linear or with nonlinear impairments under 5G channels.

This paper is an extension of J. A. Soares’ MSc. dissertation developed at the School of Electrical and Computer Engineering, University of Campinas, in the area of Telecommunications and Telematics [11]. In addition to the complex-valued RBF-based MIMO system proposed in the dissertation [11], in this paper, the MIMO-PTRBF receiver is further elaborated with additional results and with the use of 5G channel models.

The remainder of this work is organized as follows. In Section 2, a brief review of multi-antenna systems is presented. The proposed STBC coding scheme and the MIMO-PTRBF for channel estimation and symbol detection in massive MIMO-OFDM systems are presented in Section 3. In Section 4, simulation results of the MIMO-PTRBF are compared with the results obtained with the OSTBC under maximum likelihood decoding in 5G channel models. Computational complexities are presented in Section 5 and conclusions are discussed in Section 6.

## 2. Background

The main goal in multi-antenna systems is to increase the channel capacity with $M_{T}$ transmit and $M_{R}$ receive antennas by a factor of $\min(M_{T},M_{R})$ without using additional transmit power or spectral bandwidth [43]. Considering the MIMO digital communication system $r\left(k\right)=H^{T}x\left(k\right)+\eta \left(k\right)$, with transmitted signal $x\left(k\right)\in C^{M_{T}}$, received signal $r\left(k\right)\in C^{M_{R}}$, and additive white Gaussian noise (AWGN) vector $\eta \left(k\right)\in C^{M_{R}}$, the channel capacity of $H\in C^{M_{T}\times M_{R}}$ is expressed as [44]

(1) $$C=\log_{2}\left[\det\left(I_{M_{R}}+\frac{E_{s}}{M_{T}E_{0}}H^{H}R_{xx}H\right)\right],$$

in which $I_{M_{R}}$ is an $M_{R}\times M_{R}$ identity matrix, ${\left[\cdot \right]}^{T}$ is the transpose operator, ${\left[\cdot \right]}^{H}$ is the conjugate transpose operator, $E_{s}$ is the total transmitted signal power, $E_{0}$ is the AWGN power, $R_{xx}=E\left\{x\left(k\right)x^{H}\left(k\right)\right\}$ is the correlation matrix of x(k), and E{·} is the expectation operator.

However, if no channel state information (CSI) is available at the transmitter, we can assume that the channel components are equally probable. In this case, we consider that power is equally divided among the transmitting antennas, which implies $R_{xx}=I_{M_{T}}$. The capacity in such a case is then given by [11,45]

(2) $$C=\log_{2}\left[\det\left(I_{M_{R}}+\frac{E_{S}}{M_{T}N_{0}}H^{H}H\right)\right].$$

Note that Equation (2) can be outperformed if the channel information is available at the transmitter (leading to a coding gain). However, Equation (2) is the maximum diversity capacity without channel knowledge at the transmitter. Furthermore, if $M_{T}=M_{R}=1$, Equation (2) represents the Shannon capacity for SISO systems [11].

In order to increase capacity, the concepts of diversity [46], coding [47], and array [48] gains play key roles in MIMO systems. The array gain is the average increase in the signal-to-noise ratio (SNR) at the receiver that arises from the coherent combining effect of multiple antennas at the transmitter, receiver, or both. Multiple antenna systems require perfect channel knowledge at the transmitter, receiver, or both to achieve this array gain [48]. On the other hand, diversity gain is obtained by the provision of replicas of the transmitted signal at the receiver [46]. Diversity gain techniques are used to mitigate degradations in the error performance due to wireless fading channels (e.g., due to multipath). Since the probability that statistically independent fading channels simultaneously experience deep fading is insignificant, there are various ways of performing diversity gain and space diversity. To accomplish this, it is necessary to use sufficiently separated antennas in the array (by more than 10λ on base stations and 2λ to 5λ on mobile devices [44]) to guarantee independent wireless channels. In contrast, coding gain is usually provided by temporal channel coding, e.g., convolutional and block codes [11].

Space–time code is a digital communication technique used to transmit multiple copies of a data stream via multiple antennas to compensate for fading and AWGN. At the receiver side, these multiple copies of the signal are received by one or more antennas, improving the communication reliability. Depending on the encoder algorithm at the transmitter, we can have different space–time codes. Space–time trellis codes (STTCs) combine modulation and trellis coding to transmit signals over a MIMO channel. Although STTCs provide both coding gain and diversity gain, the computational complexity is higher than other space–time codes, mainly in the receiver, where a Viterbi decoder is necessary [49]. Space–time block codes (STBCs) combine multiple symbols from a digital modulation, creating a block of symbols. The components of this block (i.e., matrix of symbols) are indexed by the transmitting antenna and the transmitting time. At the transmitter, STBC decoding is performed in linear processing. Another technique is the space–time labeling diversity (STLD), a variation of STBC that takes two bit-streams and outputs two pairs of symbols. Two symbols in each pair are transmitted by two transmit antennas in two time slots, which results in full-diversity and half-rate [50]. In addition, STLD only works with a limited number of transmit antennas.

### 2.1. Space–Time Block Coding and OFDM

MIMO systems are mainly designed for narrowband or flat channels. Applying MIMO systems in the wideband frequency selective channel implies a constant penalty factor in the coding gain compared with that in flat-frequency channels. Furthermore, at high SNRs, an irreducible error rate floor is inevitable [51]. This irreducible error rate *floor* is due to the existence of multipath delay spread, and it persists even if we increase the number of antennas. Since the ISI is the root cause of the error *floor*, in principle, it can be mitigated by resorting to adaptive equalization, but this can be too complex to implement in such an environment. Another option that is widely used is to resort to OFDM, which naturally converts a frequency-selective fading channel into a frequency-nonselective fading channel. The subcarriers (i.e., tones) in an OFDM symbol are essentially narrowband signals. Since these tones fit perfectly as vehicles for space–time codes, OFDM is an enabler for this efficient coding technique [11].

**Figure 1:**
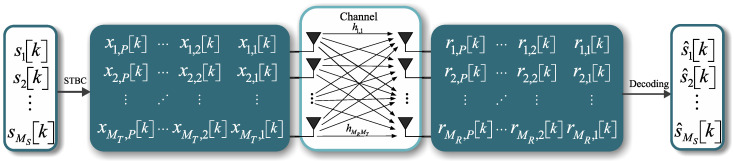
Coding scheme for a MIMO-OFDM system in which *k* is the index of the *k*th MIMO-STBC-encoded symbol matrix X[k] along the OFDM symbol [11].

Figure 1 shows the coding scheme for a generic coding matrix $X\left[k\right]\in C^{M_{T}\times P}$, where *P* is the number of time samples for the transmission of one block of coded symbols and k=1,2,⋯,K is the carrier index of the *k*th MIMO-STBC encoded symbol matrix X[k] along the OFDM symbol. $R\left[k\right]\in C^{M_{R}\times P}$ is the matrix of received symbols, $\hat{s}\in C^{M_{S}}$ is the decoded vector, and $M_{S}$ is the number of modulated symbols in a MIMO-STBC matrix.

**Figure 2:**
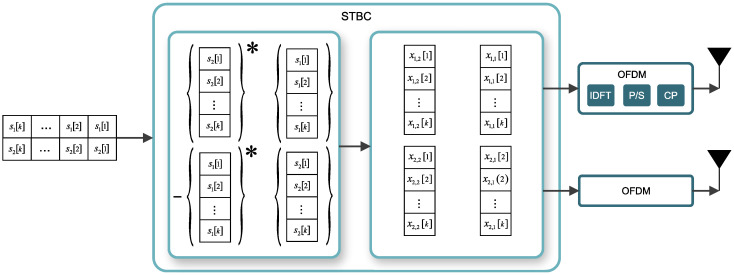
Detailed MIMO-OFDM coding system. ${\left\{\cdot \right\}}^{*}$ denotes the conjugate operator.

The transmitting space–time block coder (STBC) encodes the data symbol vector $s\left[k\right]\in C^{M_{S}}$ using the code matrix to construct the transmitting matrix X[k] of length *K*. The streams $X_{m_{T},p}\left[k\right]$ are fed to the IFFT modulator of each $m_{T}$ transmitting antenna, at each *p* period of time relative to the OFDM symbol sequence. In this manner, the information is transmitted in X[k] blocks of $M_{T}$ antennas and *P* OFDM symbols in each *k*-th carrier. To illustrate this scheme, Figure 2 shows an example for a two-transmitting antenna system using Alamouti coding. Consequently, the channel is given by $H\in C^{M_{T}\times M_{R}\times P\times K}$. It should be emphasized that in the simulation in Section 4 of this work, the channel is not assumed to be static over the entire MIMO-STBC block since it spreads over time in *P*-consecutive OFDM symbols. This is particularly necessary for the proposed work, as the receiver will fit and adapt to the characteristics and variations of the channel over time. This is also necessary for a massive number of broadcast antennas due to the length of the long block coding *P* that transmits over time in consecutive OFDM symbols [11].

**Figure 3:**
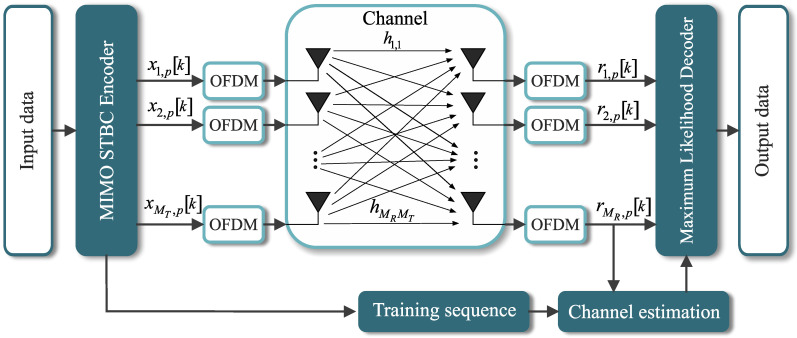
MIMO-OFDM model system with channel estimation.

As in OFDM systems, MIMO-OFDM also requires the channel state information to decode the received symbols. One of the most popular and widely used approaches to MIMO channel estimation is to employ pilot signals (also referred to as training sequences) and then estimate the channel based on the received data and the knowledge of the training sequence, as detailed in Figure 3. Based on pilot signals, in [52,53], the least-squares (LS) channel estimation technique is applied for orthogonal frequency-division multiplexing systems with multiple transmit antennas [11].

A generalized coding scheme referred to as space–time block codes (STBCs) [16,44,54], based on the theory of orthogonal matrix designs, can achieve the full-transmit diversity of $M_{T}M_{R}$ employing the maximum likelihood decoding algorithm at the receiver [44]. The idea is to transmit $M_{T}$ orthogonal streams, which implies that the receiver antennas receive $M_{T}$ orthogonal streams. This special class of space–time block codes is the so-called orthogonal STBC (OSTBC) [11,54,55].

An OSTBC example of coding matrix for $M_{T}=4$ [44] is given by

(3) $${\mathrm{OSTBC}}_{4,8}=\left[\begin{array}{cccccccc} s[1] & -s[2] & -s[3] & -s[4] & s{\left[1\right]}^{*} & -s{\left[2\right]}^{*} & -s{\left[3\right]}^{*} & -s{\left[4\right]}^{*}\\ s[2] & s[1] & s[4] & -s[3] & s{\left[2\right]}^{*} & s{\left[1\right]}^{*} & s{\left[4\right]}^{*} & -s{\left[3\right]}^{*}\\ s[3] & -s[4] & s[1] & s[2] & s{\left[3\right]}^{*} & -s{\left[4\right]}^{*} & s{\left[1\right]}^{*} & s{\left[2\right]}^{*}\\ s[4] & s[3] & -s[2] & s[1] & s{\left[4\right]}^{*} & s{\left[3\right]}^{*} & -s{\left[2\right]}^{*} & s{\left[1\right]}^{*} \end{array}\right],$$

in which $s[m_{s}]$ is the transmitted signal in the discrete symbol index $m_{s}$. Notice that, as proved by Tarokh et al. [54], the inner product of any two distinct rows of this matrix is equal to zero (i.e., the matrix is orthogonal) and of full-rank, yielding full-diversity [11].

One of the disadvantages of OSTBC is the code rate. Let *P* represent the number of time samples to convey one block of coded symbols and $M_{s}$ represent the number of symbols transmitted per block. The space–time block code rate is defined as the ratio between the number of symbols that the encoder receives at its input and the number of space–time coded symbols transmitted from each antenna, given by $R=M_{s}/P$. This implies that Equation (3) has a code rate R=1/2, which consequently reduces the spectral efficiency. Supplementary to the diversity gain, the OSTBC leads to a secondary linear coding gain $G_{c}=10\log\left(R\right)$ at the receiver due to the coherent detection of multiple copies of the signal over time. Furthermore, the multi-antenna system, as presented in Figure 1, will lead to an array gain $G_{a}=10\log\left(M_{R}\right)$ due to the coherent combination of multiple received signals over the receiving antennas [11].

### 2.2. Quasi-Orthogonal Special Case

In order to increase the spectral efficiency in orthogonal codes, Jafarkhani [56] proposed quasi-orthogonal STBC (QOSTBC) of rate one, relaxing the requirement of orthogonality. However, when compared with orthogonal codes, the diversity gain is reduced by a factor of two. Besides, in contrast to orthogonally designed codes that process one symbol at a time at the decoder, quasi-orthogonal codes process pairs of transmitted symbols, which exponentially increases the computational complexity of decoding [11].

Jafarkhani [56] proposed a coding matrix of rate one for $M_{T}=4$, given by

(4) $${\mathrm{QOSTBC}}_{4,4}=\left[\begin{array}{cccc} s[1] & s[2] & s[3] & s[4]\\ -s{\left[2\right]}^{*} & s{\left[1\right]}^{*} & -s{\left[4\right]}^{*} & s{\left[3\right]}^{*}\\ -s{\left[3\right]}^{*} & -s{\left[4\right]}^{*} & s{\left[1\right]}^{*} & s{\left[2\right]}^{*}\\ s[4] & -s[3] & -s[2] & s[1] \end{array}\right].$$

In the literature, related approaches with a maximum of $M_{T}=6$ antennas were proposed for quasi-orthogonal codes [57,58,59]. In [58], the authors developed an architecture similar to [56]; however, this presents full-diversity at the cost of more processing and is limited to $M_{T}=4$ antennas. In the same way, by increasing the decoding processing, Sindhu and Hameed [59] proposed two quasi-orthogonal schemes with $M_{T}=5$ and 6 antennas [11].

### 2.3. Decoding for Space–Time Block Codes

Maximum likelihood (ML) detection calculates the Euclidean distance among the received signal matrix R and the product of all possible transmitted signal vectors by the channel matrix H. Considering A, the set of constellation symbols of the transmitted signal, and $M_{S}$, the number of transmitted symbols per MIMO block, ML detection determines the estimation of the conveyed signal vector s as [11]

(5) $$\hat{s}=\underset{s\;\in \;A^{M_{S}}}{\mathrm{argmin}}{∥\begin{array}{c} R-H^{T}X \end{array}∥}^{2}.$$

As in maximum a posteriori (MAP) detection, ML detection achieves the optimal performance when all transmitted vectors are equally probable. However, the number of ML computation metrics is $A^{M_{S}}$, where *A* is the modulation order. Thus, the ML complexity increases exponentially with the modulation order or the number of transmit symbols, or both [44,54,60]. Although this method has a high computational complexity, the ML decoding is used as a benchmark due to its optimal performance [11].

For orthogonal coding schemes, the ML metric can be simplified, decoding symbol by symbol [54]. Via this simplification, it is possible to circumvent the issue of exponential computational complexity. However, even with this simplification, the computational complexity can be considerably high. In QOSTBC, the ML metric can be also simplified, but the computational complexity remains higher than the orthogonal case, because QOSTBC is decoded in pairs of symbols [11].

## 3. Proposed Approach

### 3.1. Coding Scheme

Similarly to the work of [56], the present work is derived from the full-rate full-diversity complex-valued space–time block code scheme proposed by Alamouti [61]. The transmission matrix proposed in [61] is given by [11]

(6) $$A_{i,j}=\left[\begin{array}{cc} s[i] & s[j]\\ -s{\left[j\right]}^{*} & s{\left[i\right]}^{*} \end{array}\right],$$

in which s[i] is the *i*th input symbol to be encoded.

Based on [61], Jafarkhani [56] proposed a quasi-orthogonal coding scheme using four antennas and consequently four encoded symbols as [11]

(7) $$S_{4}^{4}=\left[\begin{array}{cc} A_{1,2} & A_{3,4}\\ -{\left[A_{3,4}\right]}^{*} & {\left[A_{1,2}\right]}^{*} \end{array}\right]=\left[\begin{array}{cccc} s[1] & s[2] & s[3] & s[4]\\ -s{\left[2\right]}^{*} & s{\left[1\right]}^{*} & -s{\left[4\right]}^{*} & s{\left[3\right]}^{*}\\ -s{\left[3\right]}^{*} & -s{\left[4\right]}^{*} & s{\left[1\right]}^{*} & s{\left[2\right]}^{*}\\ s[4] & -s[3] & -s[2] & s[1] \end{array}\right],$$

where S is the quasi-orthogonal coding matrix. The main idea behind the work of [56] is to build a 4×4 matrix from two 2×2 matrices, keeping a fixed transmission rate [11].

In the present paper, we generalize the idea presented in [56] to a new recursive method of generating coding schemes, as given by [11]

(8) $$S_{M_{s}}^{M_{T}}=\left[\begin{array}{cc} S_{M_{s}-M_{T}/2}^{M_{T}/2} & S_{M_{s}}^{M_{T}/2}\\ -{\left[S_{M_{s}}^{M_{T}/2}\right]}^{*} & {\left[S_{M_{s}-M_{T}/2}^{M_{T}/2}\right]}^{*} \end{array}\right],$$

in which $M_{T}=2^{n},\;\forall \;n\geq 1$ is the number of transmitting antennas and $M_{s}$ is the number of encoded symbols. In the proposed scheme, $M_{s}≜M_{T}$ and the code rate is $R=M_{T}/M_{s}=1$ [11]. The recurrence is performed until we find $S_{n}^{1}=s\left[n\right],\;\forall n\in \left[1,\;2,\cdots ,M_{S}\right]$ in Equation (8).

For example, with four transmitting antennas, Equation (8) results in [11]

(9) $$S_{4}^{4}=\left[\begin{array}{cc} S_{4-4/2}^{4/2} & S_{4}^{4/2}\\ -{\left[S_{4}^{4/2}\right]}^{*} & {\left[S_{4-4/2}^{4/2}\right]}^{*} \end{array}\right]=\left[\begin{array}{cc} S_{2}^{2} & S_{4}^{2}\\ -{\left[S_{4}^{2}\right]}^{*} & {\left[S_{2}^{2}\right]}^{*} \end{array}\right].$$

From the recurrent structure of Equation (8), $S_{2}^{2}$ and $S_{2}^{4}$ are

(10) $$S_{2}^{2}=\left[\begin{array}{cc} S_{1}^{1} & S_{2}^{1}\\ -{\left[S_{2}^{1}\right]}^{*} & {\left[S_{1}^{1}\right]}^{*} \end{array}\right],$$

and

(11) $$S_{4}^{2}=\left[\begin{array}{cc} S_{3}^{1} & S_{4}^{1}\\ -{\left[S_{4}^{1}\right]}^{*} & {\left[S_{3}^{1}\right]}^{*} \end{array}\right].$$

Thus, substituting Equations (10) and (11) into Equation (9),

(12) $$S_{4}^{4}=\left[\begin{array}{cc} \left[\begin{array}{cc} S_{1}^{1} & S_{2}^{1}\\ -{\left[S_{2}^{1}\right]}^{*} & {\left[S_{1}^{1}\right]}^{*} \end{array}\right]\; & \left[\begin{array}{cc} S_{3}^{1} & S_{4}^{1}\\ -{\left[S_{4}^{1}\right]}^{*} & {\left[S_{3}^{1}\right]}^{*} \end{array}\right]\\ -{\left[\begin{array}{cc} S_{3}^{1} & S_{4}^{1}\\ -{\left[S_{4}^{1}\right]}^{*} & {\left[S_{3}^{1}\right]}^{*} \end{array}\right]}^{*}\; & {\left[\begin{array}{cc} S_{1}^{1} & S_{2}^{1}\\ -{\left[S_{2}^{1}\right]}^{*} & {\left[S_{1}^{1}\right]}^{*} \end{array}\right]}^{*} \end{array}\right]=\left[\begin{array}{cccc} S_{1}^{1} & S_{2}^{1} & S_{3}^{1} & S_{4}^{1}\\ -{S_{2}^{1}}^{*} & {S_{1}^{1}}^{*} & -{S_{4}^{1}}^{*} & {S_{3}^{1}}^{*}\\ -{S_{3}^{1}}^{*} & -{S_{4}^{1}}^{*} & {S_{1}^{1}}^{*} & {S_{2}^{1}}^{*}\\ S_{4}^{1} & -S_{3}^{1} & -S_{2}^{1} & S_{1}^{1} \end{array}\right].$$

Replacing $S_{n}^{1}=s\left[n\right],\;\forall n\in \left[1,\;2,\;3,\;4\right]$, into Equation (12),

(13) $$S_{4}^{4}=\left[\begin{array}{cccc} s[1] & s[2] & s[3] & s[4]\\ -s{\left[2\right]}^{*} & s{\left[1\right]}^{*} & -s{\left[4\right]}^{*} & s{\left[3\right]}^{*}\\ -s{\left[3\right]}^{*} & -s{\left[4\right]}^{*} & s{\left[1\right]}^{*} & s{\left[2\right]}^{*}\\ s[4] & -s[3] & -s[2] & s[1] \end{array}\right].$$

Note that Equation (13) is equal to the coding scheme proposed by [56] with four antennas, as in Equation (4). However, in contrast to the work of [56], our coding scheme, presented in Equation (8), can generate coding matrices for any $M_{T}=2^{n},\;\forall \;n\geq 1$, and $M_{s}≜M_{T}$ [11]. For the case of n=1, Equation (8) is equal to Equation (6), the full-rate full-diversity complex-valued space–time block code scheme proposed in [61].

The main issue of the proposed coding scheme is that we cannot define a simplified ML decoding method as in the former cases. Then, it is here that the system proposed in this paper takes shape, with the MM-PTRBF decoding, making the joint solution feasible. We have observed, by extensive simulations, that Equation (8) achieves half of the diversity presented by the orthogonal coding schemes but keeps full-rate (i.e., R=1), which is essentially the characteristics of the quasi-orthogonal scheme proposed by [11,56].

### 3.2. Complex MIMO-PTRBF Neural Network for Massive MIMO Decoding

**Figure 4:**
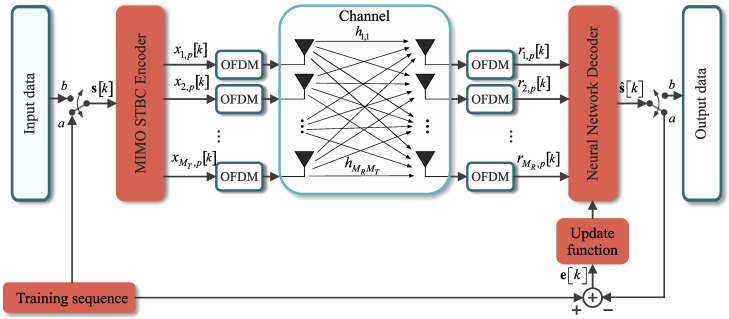
Complete vision of the proposed MIMO-OFDM model [11].

In the proposed system, the maximum likelihood decoder is replaced by a neural network, the MIMO-PTRBF, to decode the received symbols, as shown in Figure 4. The MIMO-PTRBF has a supervised learning stage, in which a training sequence is used to fit the hyper-parameters of the neural network. A pseudo-random generator creates this training sequence, which is known both at the transmitter and receiver sides. When the neural network output achieves the desired MSE, it switches from the learning stage to the decoding stage. At this time, the information data are then effectively transmitted over the system, and the BER is computed. These two stages are implemented as in Figure 4, with the input switch of the MIMO STBC Encoder block and the output switch of the Neural Network Decoder block. The switches have two states represented by (a) and (b), which shift between the training and decoding stages [11].

**Figure 5:**
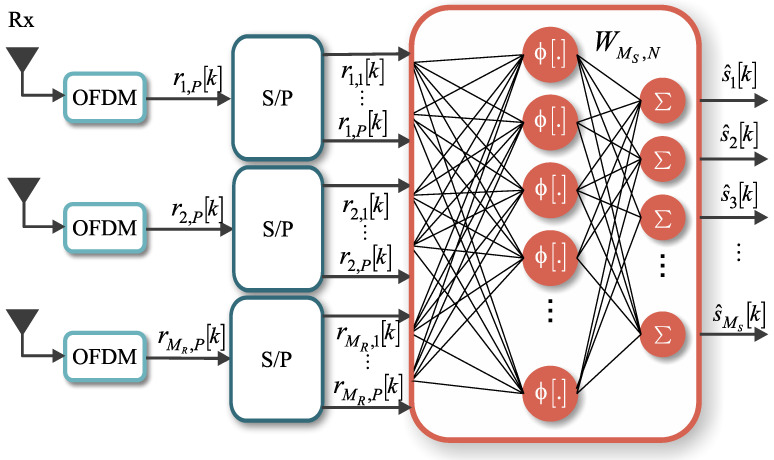
Closer view of the system with neural network decoding [11].

As in the maximum likelihood detector, the input signal to the MIMO-PTRBF algorithm is the set of received vectors r, as shown in Figure 4. The MIMO-PTRBF architecture, with *N* neurons, has three free parameters: the matrix of synaptic weights $W\in C^{M_{s}\times N}$, the matrix of center vectors $\Gamma \in C^{M_{R}P\times N}$, and the vector of variances $\sigma^{2}\in C^{N\times 1}$. The MIMO-PTRBF is an extension of the PTRBF for multiple outputs. The key difference between both architectures is the multiple-output layer, which fits each output individually. Figure 5 shows a closer view of the receiver side using the MIMO-PTRBF neural network for decoding [11].

The output vector is thus given by

(14) $$\hat{s}\left[u\right]=W\left[u\right]\phi \left[u\right].$$

Following the complex-valued radial basis function presented in [41], the *n*th neuron output of the MIMO-PTRBF ($\phi_{n}$), for the *p*th output vector of r, is [11]

**Figure 6:**
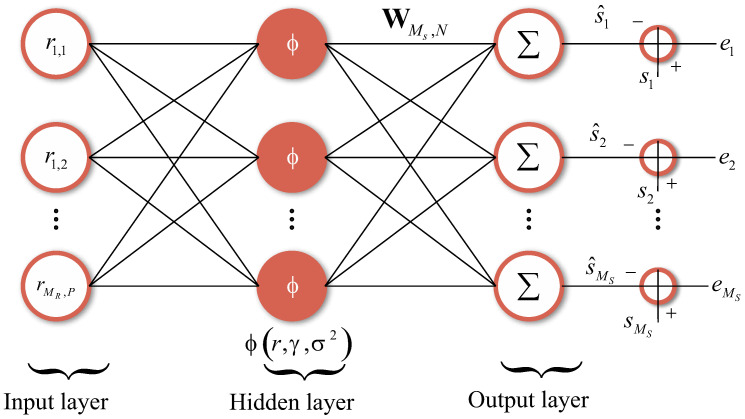
MIMO phase transmittance radial basis function neural network architecture [11].

(15) ϕn=exp−||Re{r}−Re{γn}||22Re{σn2}+jexp−||Im{r}−Im{γn}||22Im{σn2},

where $\left|\right|\cdot {\left|\right|}_{2}$ is the operator which returns the Euclidean norm of its argument, and Re{·} and Im{·} are the respective real and imaginary parts of their arguments. Additionally, as shown in Figure 6, the output of the neurons can be represented by the vector $\phi ={\left[\phi_{1}\;\phi_{2}\;\cdots \;\phi_{N}\right]}^{T}\in C^{N\times 1}$. This kernel partitioning into real and imaginary components has an important role in avoiding any phase invariance at the output of the neurons [11,35,41].

Thus, by means of the steepest descent algorithm, the update of the MIMO-PTRBF free parameters is given by

$$w_{m_{s},n}\left[u+1\right]=w_{m_{s},n}\left[u\right]-\eta_{w}\nabla^{w}J\left[u\right],$$

(16) $$\gamma_{n}\left[u+1\right]=\gamma_{n}\left[u\right]-\eta_{\gamma }\nabla^{\gamma }J\left[u\right],$$

$$\sigma_{n}^{2}\left[u+1\right]=\sigma_{n}^{2}\left[u\right]-\eta_{\sigma }\nabla^{\sigma }J\left[u\right],$$

in which $\eta_{w}$, $\eta_{\gamma }$, and $\eta_{\sigma }$ are the adaptive steps of $w_{m_{s},n}$, $\gamma_{n}$, and $\sigma_{n}^{2}$, respectively. Furthermore, $\nabla^{w}$, $\nabla^{\gamma }$, and $\nabla^{\sigma }$ are the complex gradient operators of $w_{m_{s},n}$, $\gamma_{n}$, and $\sigma_{n}^{2}$, respectively.

Thus, with r and s, the MIMO-PTRBF algorithm can be used to estimate the output vector $\hat{s}$ at the *u*th training epoch by the minimization of the following cost function:

(17) $$J\left[u\right]=\frac{1}{2}||s\left[u\right]-\hat{s}\left[u\right]{\left|\right|}_{2}^{2},$$

where s and $\hat{s}$ are the training sequence and the output vector, respectively.

Applying the complex gradient operators ($\nabla^{w}$, $\nabla^{\gamma }$, and $\nabla^{\sigma }$) to (17) yields

$$\nabla^{w}J\left[u\right]=-e_{m_{s}}\left[u\right]\phi_{n}^{*}\left[u\right],$$

(18) ∇γJn[u]=−ξ*[u]ωn[u]Re{αn[u]}−Im{αn[u]}−ξ[u]ωn*[u]Re{αn[u]}+Im{αn[u]},

∇σJn[u]=−ξ*[u]ωn[u](Re{βn[u]}−Im{βn[u]})−ξ[u]ωn*[u](Re{βn[u]}+Im{βn[u]}),

in which $e_{m_{s}}\left[u\right]=s_{m_{s}}\left[u\right]-{\hat{s}}_{m_{s}}\left[u\right]$ is the instantaneous error for the output ${\hat{s}}_{m_{s}}$ at the *u*th training epoch. Then, substituting Equation (18) in (16) yields

(19) wms,n[u+1]=wms,n[u]+ηwems[u]ϕn*[u],γn[u+1]=γn[u]+ηγRe(ξn[u])Re(αn[u])−jIm(ξn[u])Im(αn[u]),σn[u+1]=σn[u]+ησRe(ξn[u])[Re(βn[u])−jIm(ξn[u])Im(βn[u]),

in which ${\left[\cdot \right]}^{*}$ denotes the complex conjugate operator and $\xi_{n}\left[u\right]$ is the *n*th synaptic transmittance, given by

(20) $$\xi_{n}\left[u\right]=\sum_{m_{s}=1}^{M_{s}}e_{m_{s}}^{*}\left[u\right]w_{m_{s},n}\left[u\right].$$

Furthermore, $\alpha_{n}\left[u\right]\in C^{R}$ is the *m*th vector of the matrix of weighted centers ($A\left[u\right]\in C^{N\times R}$):

(21) αn[u]=Re(ϕn[u])Re(x[u])−Re(γn[u])Re(σn[u])+jIm(ϕn[u])Im(x[u])−Im(γn[u])Im(σn[u]).

In a similar way, $\beta_{n}\left[u\right]\in C$ is the *n*th element of the vector of weighted kernel ($\beta \left[u\right]\in C^{N}$):

(22) βn[u]=Re(ϕn[u])Re(x[u])−Re(γn[u])22Re(σn[u])2+jIm(ϕn[u])Im(x[u])−Im(γn[u])22Im(σn[u])2.

Generalizing Equation (19) to matrix structures results in

(23) $$\begin{array}{c} W\left[u+1\right]=W\left[u\right]+\eta_{w}e\left[u\right]\phi^{H}\left[u\right],\\ \Gamma \left[u+1\right]=\Gamma \left[u\right]+\eta_{\gamma }{Æ}^{*}\left[u\right],\\ \sigma \left[u+1\right]=\sigma \left[u\right]+\eta_{\sigma }{æ}^{*}\left[u\right], \end{array}$$

where Æ[u] and æ[u] are auxiliary variables used to reduce the computational complexity. Æ[u] and æ[u] are given by

(24) Æ[u]=Re(Ξ[u])Re(A[u])+jIm(Ξ[u])Im(A[u])∈CN×R,æ[u]=Re(Ξ[u])Re(β[u])+jIm(Ξ[u])Im(β[u])∈CN,

in which Ξ[u] is the diagonal matrix of synaptic transmittance:

(25) $$\Xi \left[u\right]=\left[\begin{array}{cccc} \xi_{1}\left[u\right] & 0 & \cdots & 0\\ 0 & \xi_{2}\left[u\right] & \cdots & 0\\ \vdots & \vdots & \ddots & \vdots \\ 0 & 0 & \cdots & \xi_{N}\left[u\right] \end{array}\right]\;\in \;C^{N\times N}.$$

Each training update is given by Equation (23); however, for u=0, the MIMO-PTRBF free parameters are initialized following some criterion defined by the user (e.g., based on the probability distribution of the input data). Although (23) minimizes the error between the output vector $\hat{s}$ and the reference vector s, as the neurons are dependent on exponential functions, a risk of instability is assumed if the exponential argument is positive. In order to circumvent this issue, based on Theorem A1 of [35], the real and imaginary parts of each scalar component of the vector of variances are lower-bounded by the limit μ>0, which, consequently, bounds the real and imaginary parts of the neurons output from 0 to 1 [11]. In addition, taking into account Theorem A2 (see Appendix A), the adaptive step of the matrix of synaptic weights is limited by $\eta_{w}<1/N$ for all simulations, to guarantee convergence in the mean. In addition, in the Appendix, Corollaries A1 and A2 are of utmost importance to prove Theorem A1. In addition, Definition A1 is used to prove Corollary A2.

## 4. Simulation Results

**Figure 7:**
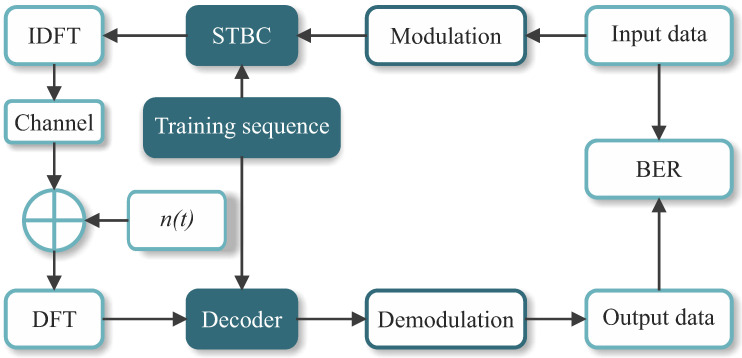
Block model of the simulated systems.

Using the formerly mentioned OSTBC [54] and QOSTBC [56] coding schemes, several setups are compared with the proposed approach to validate and assess their performance in massive MIMO-OFDM. OSTBC and QOSTBC are simulated with the maximum likelihood (ML) decoding with perfect channel knowledge. This configuration achieves the maximum diversity gain $G_{d}=M_{T}M_{R}$ at the cost of half of the theoretical bandwidth efficiency, since R=1/2 in this case. Considering a practical QOSTBC application, we also implement the EQOSTBC with the least-squares (ML-LS) channel estimation [11]. Figure 7 illustrates the simulated system.

In Figure 7, the binary input data are created by a pseudo-random generator with uniform distribution. The bit-stream is then modulated according to the *M*-QAM or *M*-PSK modulation scheme used in the simulation. Subsequently, using the coding scheme proposed in Section 3, the modulated symbols are encoded in the STBC block. In the IDFT block, the STBC symbols are frequency-multiplexed for OFDM transmission. At the receiver side, after the transmitted signal passes through the channel, the DFT is applied to demultiplex the STBC symbols. In the decoder, the proposed ANN-based technique presented in Section 3 and the ML algorithm (either with perfect channel knowledge at the receiver or with channel estimation by LS) are employed to assess the system performance. In the sequel, the decoder output symbols are demodulated, and BER is computed.

For the sake of comparison, the BER as a function of $E_{b}/N_{0}$ (energy per bit to noise power spectral density ratio) is used in the simulations. By adjusting the transmitting power for each antenna, the received signals are normalized by $M_{T}$ transmitting antenna, by the receiver array gain $M_{R}$, and by the code rate gain *R*, implying

(26) $$SNR_{\left(dB\right)}={E_{b}/N_{0}}_{\left(dB\right)}+10\log_{10}\left(\frac{b}{RM_{T}M_{R}}\right),$$

in which *b* is the number of bits per QAM symbol.

**Figure 8:**
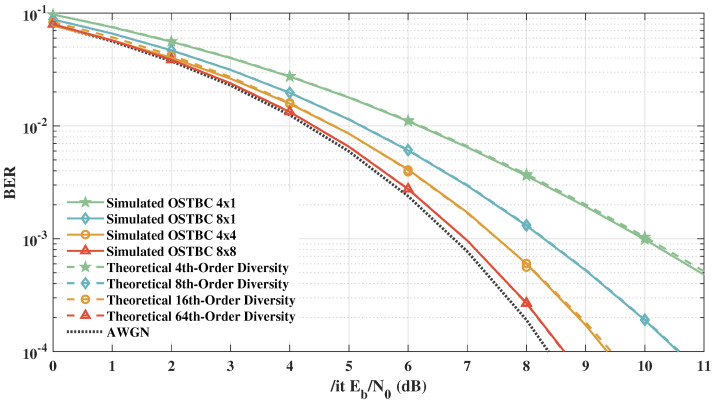
Simulated and theoretical results for 4th, 8th, 16th, and 64th diversity orders.

**Figure 9:**
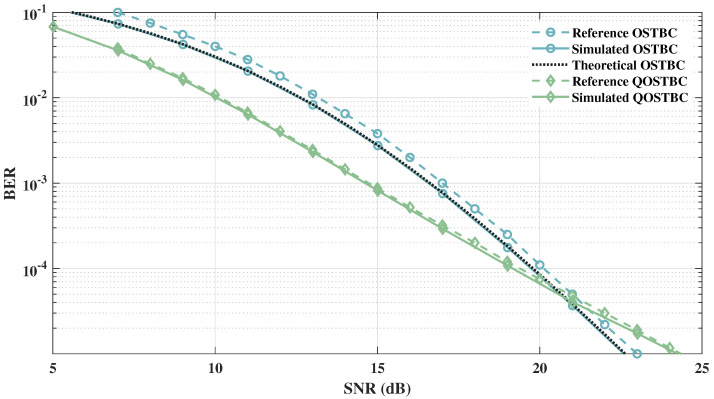
Simulated, reference, and theoretical results for equal diversity order and bitrate [11].

In Figure 8, aiming to validate the simulator shown in Figure 7, we compare the obtained results with the theoretical performance of OSTBC for 4th, 8th, 16th, and 64th diversity orders using 4-QAM modulation for a Rayleigh channel with AWGN. For all OSTBC diversity orders, theoretical and simulated results were approximately the same, validating the framework. In addition, Figure 9 presents the reference results of [56] with $M_{T}=4$ antennas and $M_{R}=1$ antenna for 16-QAM OSTBC and 4-QAM QOSTBC and the obtained results for the same scenarios. The simulated results are in line with theoretical results, which also corroborates the framework’s reliability.

**Figure 10:**
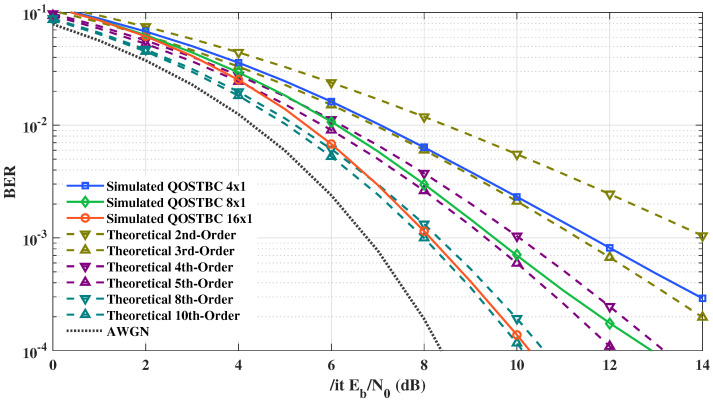
Simulation for the proposed coding scheme against theoretical results for equal bitrate and $M_{T}=4$ and 8 with $M_{R}=1$ [11].

With the simulation framework validated, we can compare the proposed coding algorithm with results from the literature. Firstly, Figure 10 shows the results of the proposed coding algorithm and theoretical results for 2nd, 3rd, 4th, 5th, 8th, and 10th order diversity. As addressed by [56], quasi-orthogonal transmitting schemes with four antennas achieve at least half of the theoretical diversity ($D_{o}=\frac{M_{T}}{2}=2$) of the orthogonal four antenna scheme ($D_{o}=M_{T}=4$). This can be seen in the solid blue curve with squares of Figure 10, which is located between the theoretical second and third-order curves. Using simulations, we extend the concept introduced by [56] for QOSTBC with 8×1 and 16×1 antennas. In order to simulate these scenarios, we employ the proposed coding algorithm presented in Equation (8). As expected, the solid green curve with diamonds and the solid orange curve with circles are between the theoretical 4th and 5th order and the 8th and 10th order, respectively. Then, utilizing this analysis, we validate the proposed code algorithm and show that it is a suitable approach for generating QOSTBC matrices for at least 16 antennas. Higher-order QOSTBC architectures using Equation (8) are not simulated because of the extensive time required to perform maximum likelihood detection.

In order to represent more practical scenarios, we set the simulation system with a 3GPP TS 38.211 specification [62] for 5G Physical channels and modulation. The Subcarrier Spacing (Δf) scales from 15 kHz to 240 kHz. The number of active carriers is 256, and the pilot sample rate (when applicable) is $M_{T}\times 8\times f_{Doppler}$ with the conventional block-based pilot scheme [63]. We perform simulations in the extremes to demonstrate the robustness of the proposed approach.

The radio channel realizations are created using the 3GPP TR 38.901 report on 5G: Study on channel model for frequencies from 0.5 GHz to 100 GHz [64]. The 3GPP channel models [64] are applicable for frequency bands in the range of 0.5 GHz to 100 GHz. From Tapped Delay Line (TDL) models in [64], TDL-B is selected from Table 7.7.2-2 (depicted in Table 1) for the channel model simulated in this work.

In Table 1, as the channel model delays are normalized, they need to be scaled according to a desired delay spread in nanoseconds (ns):

(27) $$\tau_{scaled}=\tau_{model}DS_{ns}$$

in which $\tau_{model}$ is the normalized delay value of the TDL model, $\tau_{scaled}$ is the new delay value (in [ns]), and $DS_{ns}$ is the desired delay spread (in [ns]). From Table 7.7.3-1 [64], examples of scaling delay spreads are very short ($DS_{ns}=10$ ns), short ($DS_{ns}=30$ ns), nominal ($DS_{ns}=100$ ns), long ($DS_{ns}=300$ ns), and very long ($DS_{ns}=1000$ ns). In this work, we use a delay spread of $DS_{ns}=50$ ns.

From the channel model of Table 1, a Rayleigh distribution is used to compute each sub-channel of $H\in C^{M_{T}\times M_{R}}$ (MIMO channel matrix). The *M*-QAM BER figure for the AWGN channel is also used to define a lower bound on BER vs. $E_{b}/N_{0}$ performance. Additionally, it is assumed that all received signals are uncorrelated [11].

A realistic scenario to assess the performance of a MIMO-OFDM system must also include the nonlinear effects of the transmitter power amplifiers [11]. This is necessary because the OFDM signal can have relatively high peak values (i.e., high PAPR) in the time domain since many subcarrier components are added via an IFFT operation. A high PAPR is one of the most detrimental aspects of the OFDM system, as it decreases the SQNR (signal-to-quantization noise ratio) of ADCs (analog-to-digital converters) and DACs (digital-to-analog converters), while also imposing a back-off that degrades the efficiency of the power amplifier in the transmitter. The PAPR issue is usually more critical in the uplink since the efficiency of the power amplifier is critical due to the limited battery power in a mobile terminal. For this purpose, from now on, the results assume mild amplifier nonlinearities, represented by a first-grade power amplifier or an appropriate back-off operating point [11]. Based on [41], the nonlinearity vector $\rho ={\left[\rho_{1}\;\rho_{2}\;\rho_{3}\right]}^{T}={\left[0.9\;0.1\;0.05\right]}^{T}$ implies 90%, 10%, and 5% first, second, and third-order coefficients, respectively.

**Figure 11:**
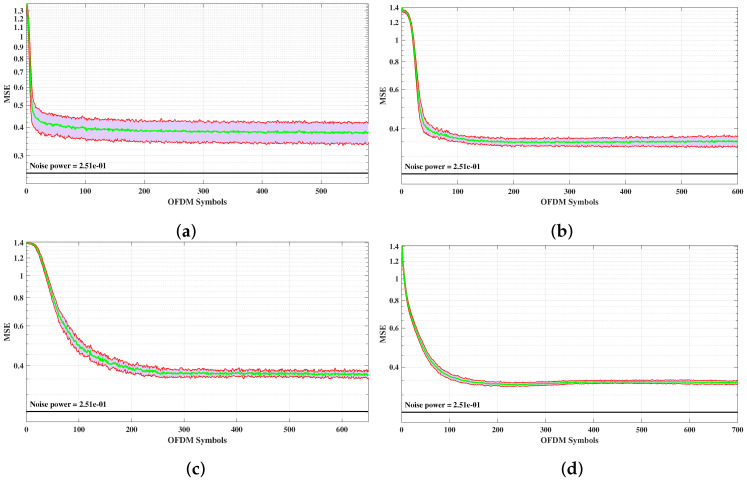
Evolution of MSE values averaged over 10 realization sequences of the proposed MMPTRBF network decoder, using 4-QAM and $E_{b}/N_{0}$ = 12 dB for (**a**) $M_{T}=M_{R}=4$ antennas, (**b**) $M_{T}=M_{R}=8$ antennas, (**c**) $M_{T}=M_{R}=16$ antennas, (**d**) $M_{T}=M_{R}=32$ antennas.

With the model properly validated and the specified 5G channel model, we are now able to analyze the proposed complex-valued ANN-based decoder for MIMO-OFDM systems. First, we present the MSE convergence curves during the learning process. The MSE curves are averaged over 10 subsequent simulation traces, and a 4-QAM modulation with $E_{b}/N_{0}=12$ dB is employed. Figure 11a–d show the MSE evolution for $M_{T}=M_{R}=4$, $M_{T}=M_{R}=8$, $M_{T}=M_{R}=16$, and $M_{T}=M_{R}=32$ antennas, respectively. The red bottom and top curves in Figure 11 refer to the MSE standard deviations over the 10 subsequent simulation traces, and the green curves refer to the mean values. Although the steady-state MSEs decrease slightly as the number of antennas increases, one may notice that the decays of the standard deviations are more conspicuous as $M_{T}=M_{R}$ increases. This is due to the MIMO characteristics that mitigate the channel effects by sending several samples of the same signal to the receiver. Thus, sudden channel variations are smoothed, suggesting that the PTRBF learning process presents a robust and cohesive behavior.

**Figure 12:**
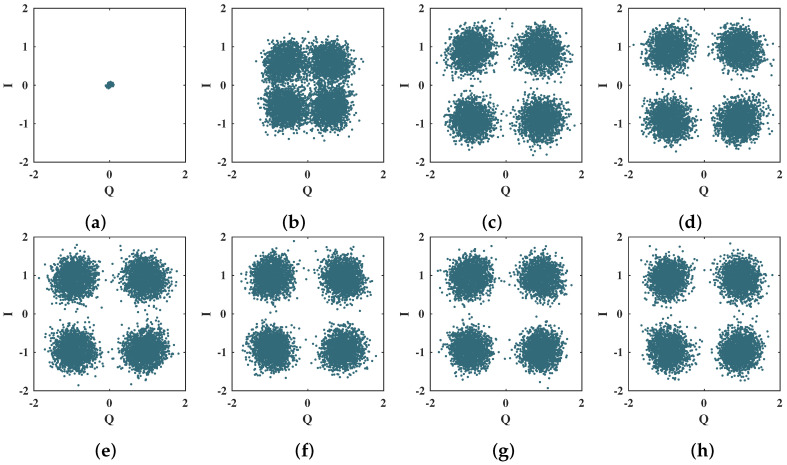
Scatter plots for the 4-QAM symbols at the output of the MMPTRBF during the training period, for $M_{T}=M_{R}=4$ antennas and $E_{b}/N_{0}$ = 12 dB. (**a**) 1 epoch, (**b**) 5 epochs, (**c**) 10 epochs, (**d**) 15 epochs, (**e**) 20 epochs, (**f**) 25 epochs, (**g**) 30 epochs, and (**h**) 35 epochs.

The 4-QAM scatter plots presented in Figure 12 show the convergence of the proposed neural network decoder for the first 35 training epochs. For this sequence of scatter plots, each training epoch corresponds to one OFDM symbol; i.e., 256 4-QAM symbols. As shown in Figure 12, the proposed algorithm has a fast convergence rate since only 10 training epochs are sufficient to separate the 4-QAM constellation symbols efficiently [11].

**Figure 13:**
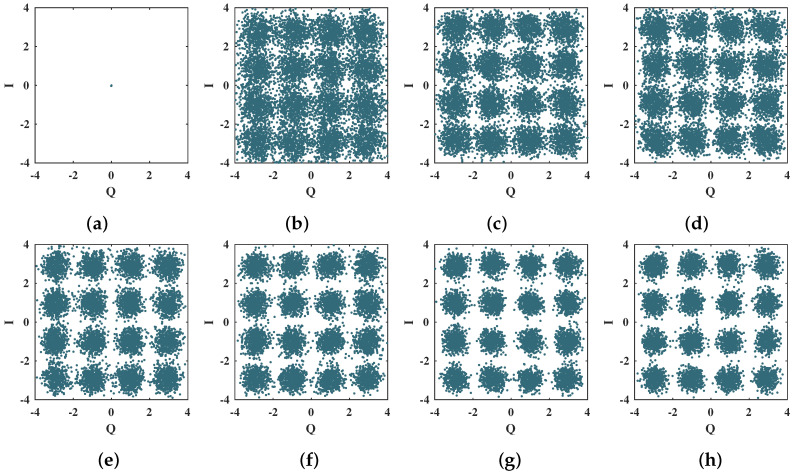
Scatter plots for the 16-QAM symbols at the output of the MMPTRBF during the training period, for $M_{T}=M_{R}=4$ antennas and $E_{b}/N_{0}$ = 20 dB. (**a**) 1 epoch, (**b**) 20 epochs, (**c**) 40 epochs, (**d**) 60 epochs, (**e**) 80 epochs, (**f**) 100 epochs, (**g**) 120 epochs, and (**h**) 140 epochs.

The 16-QAM scatter plots presented in Figure 13 show the convergence of the proposed neural network decoder for the first 140 training epochs, spaced in intervals of 20 OFDM symbols. In this case, the number of training epochs necessary for algorithm convergence is greater than for the 4-QAM case, given the intrinsic complexity of the higher-order constellation. Nevertheless, with only 20 training epochs, it is already possible to visually identify the 16-QAM constellation symbols and, with 80 training epochs, to see the correctly grouped symbols in the scatter plot [11].

**Figure 14:**
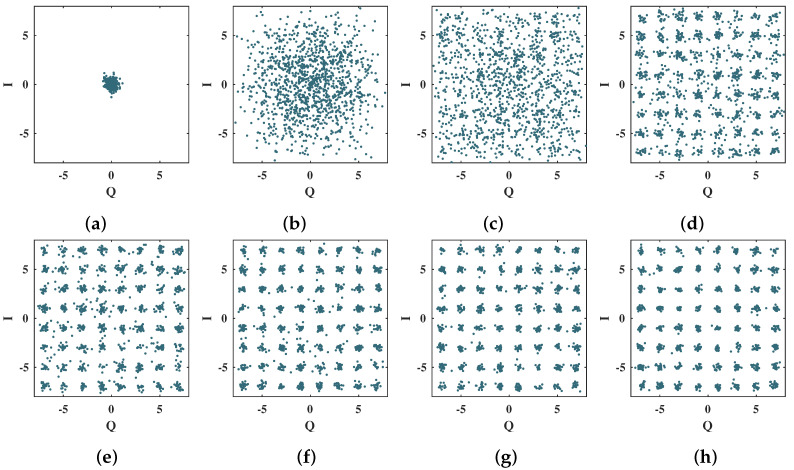
Scatter plots for the 64-QAM symbols at the output of the MMPTRBF in the training phase, for $M_{T}=M_{R}=4$ antennas and $E_{b}/N_{0}$ = 26 dB. (**a**) 1 epoch, (**b**) 1000 epochs, (**c**) 2000 epochs, (**d**) 3000 epochs, (**e**) 4000 epochs, (**f**) 5000 epochs, (**g**) 6000 epochs, and (**h**) 7000 epochs.

The 64-QAM scatter plots presented in Figure 14 show the convergence of the proposed neural network decoder for the first 7000 training epochs, spaced in intervals of 1000 OFDM symbols. In this case, the number of training epochs necessary for algorithm convergence is greater than for the former 16/4-QAM cases, in view of the intrinsic complexity of the much higher-order 64-QAM constellation. Nevertheless, with 3000 training epochs, it is already possible to visually identify the 64-QAM constellation symbols and, with 5000 training epochs, to see the correctly grouped symbols in the scatter plot [11]. Although an abrupt increase in training epochs occurs, when compared with 4-QAM in Figure 12, it represents a time interval of only 80 ms (5000 OFDM symbols with 240 kHz sub-carrier spacing).

**Figure 15:**
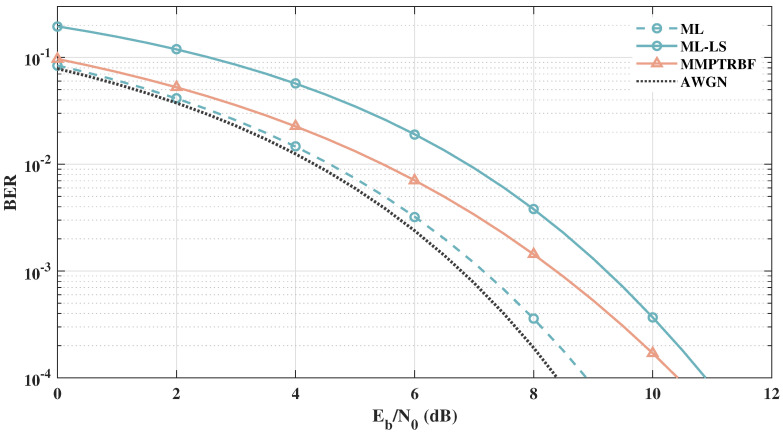
Systems with $M_{T}=M_{R}=4$ antennas for 4-QAM modulation.

After analyzing the MSE curves and constellations of the MMPTRBF, we can further investigate the BER vs. $E_{b}/N_{0}$ of the proposed approach. Figure 15 shows the BER vs. $E_{b}/N_{0}$ results of the QOSTBC, EQOSTBC, and MIMO-PTRBF systems operating with $M_{T}=M_{R}=4$ antennas and 4-QAM modulation. The 4-QAM AWGN curve defines a lower bound for all MIMO systems that use 4-QAM modulation. The simulation results in Figure 15 indicate that the QOSTBC system outperforms the proposed work when perfect channel knowledge is available at the receiver, which is impractical. Although the EQOSTBC system is a feasible and practical version of the QOSTBC, due to the channel estimation block at the receiver, simulations using the least-squares channel estimation show that the EQOSTBC performance is degraded by more than 2.5 dB when compared with the QOSTBC [11]. Furthermore, even with a perfect channel estimator, it is computationally expensive to decode QOSTBC codes with maximum likelihood for more than four antennas, as addressed by [56].

**Figure 16:**
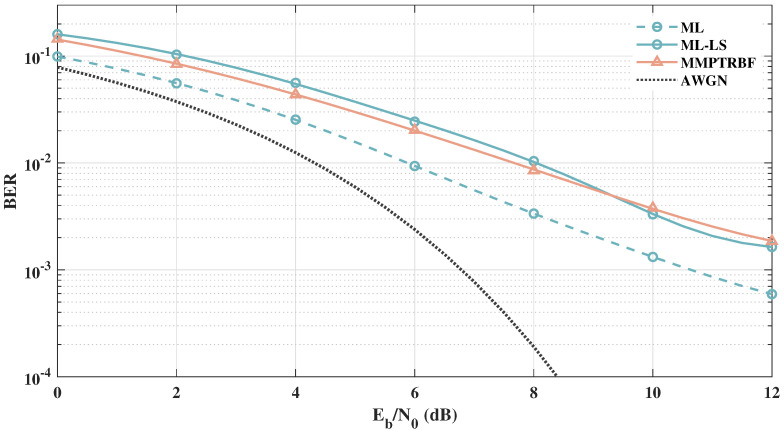
Systems with $M_{T}=4$ and $M_{R}=1$ antennas for 4-QAM modulation.

**Figure 17:**
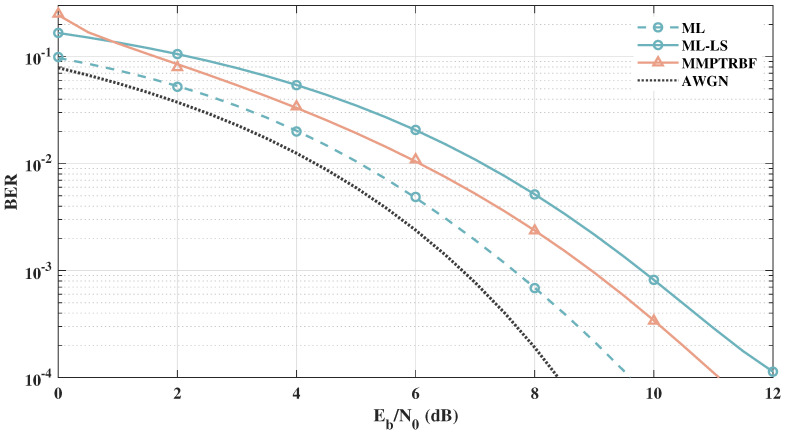
Systems with $M_{T}=8$ and $M_{R}=1$ antennas for 4-QAM modulation.

Figure 16 and Figure 17 show the BER$\times E_{b}/N_{0}$ results of the QOSTBC, EQOSTBC, and MIMO-PTRBF systems operating with $M_{T}=4$ and $M_{T}=8$ with $M_{R}=1$ antennas and 4-QAM modulation. Figure 16 and Figure 17 highlight the diversity gain of the proposed system when compared with the EQOSTBC. Although the mathematical derivation of the proposed system diversity gain has not been obtained yet, simulations indicate a significant diversity gain [11]. Contrasting Figure 15 with Figure 16, one can see the increase in the diversity gain as the number of transmitting antennas increases.

**Figure 18:**
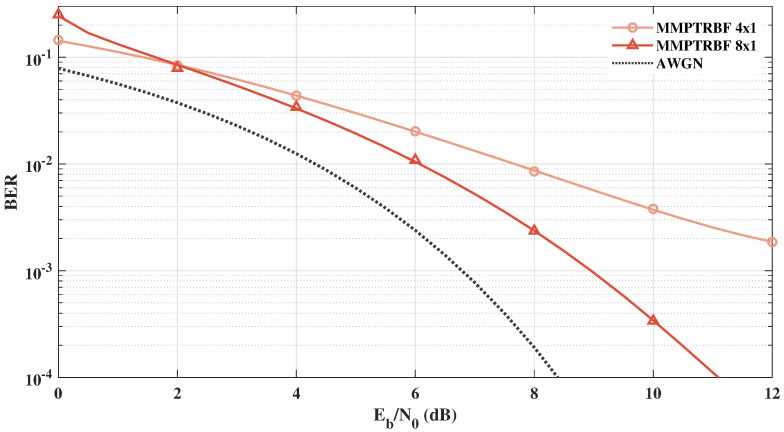
MMPTRBF with $M_{T}=4$, $M_{T}=8$, and $M_{R}=1$ antennas for 4-QAM modulation.

Figure 18 shows the BER vs. $E_{b}/N_{0}$ results of the MMPTRBF systems operating with $M_{T}=4$, 6, and 8 antennas for a 4-QAM modulation. Figure 18 also highlights the increase in the diversity gain as the number of transmitting antennas increases. For a BER $=10^{-2}$, there is a gain of approximately 2 dB when doubling $M_{T}$.

**Figure 19:**
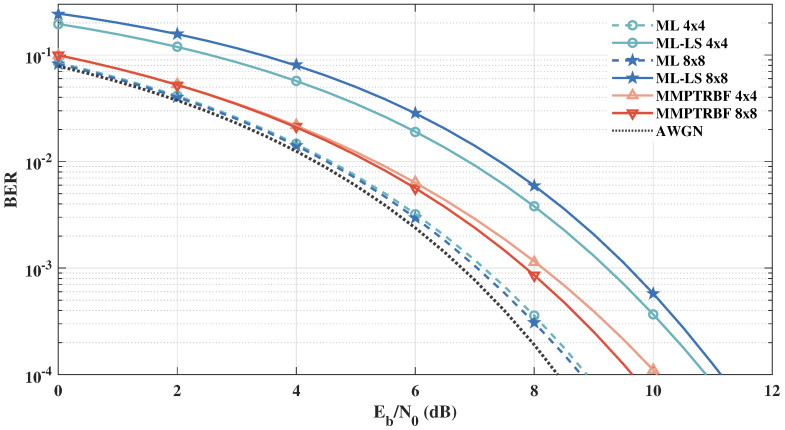
Systems with $M_{T}=M_{R}=4$, $M_{T}=M_{R}=8$ antennas for 4-QAM modulation.

To further investigate the effects of a larger number of transmitting and receiving antennas on the performances of BER, in Figure 19, we present the simulation results for a higher-order system with $M_{T}=M_{R}=8$. It can be seen that the performance of the quasi-orthogonal code with channel estimation is worse for $M_{T}=8$ than for $M_{T}=4$ antennas, as shown in Figure 19. It is shown in [53] that the performance of the linear estimator decreases proportionally with the number of transmitting antennas, which adds a constraint to the number of transmitting antennas for linearly decoded systems.

**Figure 20:**
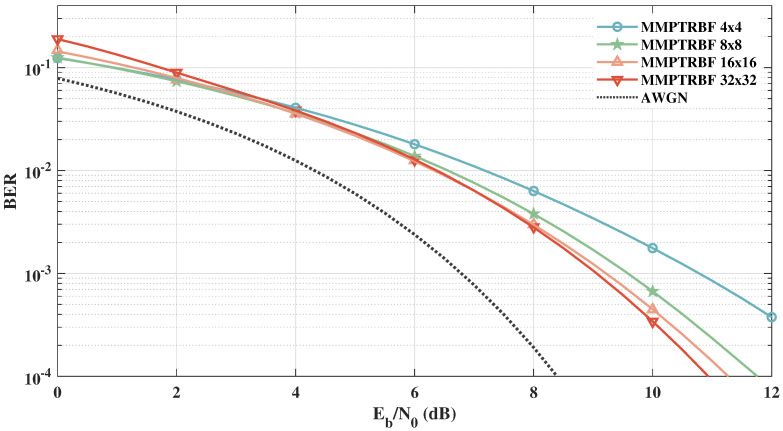
MMPTRBF with $M_{T}=M_{R}=4$, $M_{T}=M_{R}=8$, $M_{T}=M_{R}=16$, $M_{T}=M_{R}=32$, antennas for 4-QAM modulation.

Figure 20 presents the simulation results for $M_{T}=M_{R}=4$ (N=100), $M_{T}=M_{R}=8$ (N=150), $M_{T}=M_{R}=16$ (N=200), and $M_{T}=M_{R}=32$ (N=600) to examine the extent of the proposed work for massive MIMO operations. These results show the potential of the proposed work to efficiently operate with a massive number of transmitting and receiving antennas. In addition, taking as reference the BER $=10^{-3}$, one can notice that the gain increment is reduced when doubling the number of antennas as $M_{T}$ increases. For instance, increasing from $M_{T}=M_{R}=4$ to $M_{T}=M_{R}=8$ yields a gain of 1.22 dB, while increasing from $M_{T}=M_{R}=16$ to $M_{T}=M_{R}=32$ yields a gain of only 0.2 dB.

It is important to highlight that, in contrast to the maximum likelihood detection, the proposed MMPTRBF decoding is able to operate with more than 16 antennas due to its reduced computational complexity, as discussed below in Section 5.

**Figure 21:**
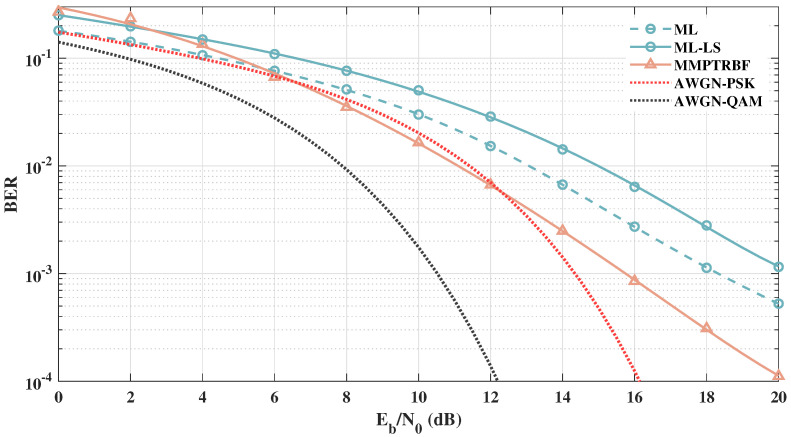
Systems with $M_{T}=M_{R}=4$ antennas operating at the same bitrate with 16-QAM (MMPTRBF) and 16-PSK (ML-QOSTBC and ML-LS-QOSTBC).

Figure 21 shows the BER vs. $E_{b}/N_{0}$ results of the ML-QOSTBC and ML-LS-QOSTBC with 16-PSK and MIMO-PTRBF with 16-QAM to further examine the extent of the proposed work for higher-order modulations with $M_{T}=M_{R}=4$ antennas. This result shows that the proposed work operates efficiently with a higher modulation order of 16-QAM. It is important to note, however, that different modulation formats are used in this scenario because the maximum likelihood QOSTBC decoding is not capable of dealing with quadrature amplitude modulation, as addressed in [56]. For this reason, in order to keep 16-order modulation, a 16-PSK modulation format for QOSTBC is used. Figure 21 shows that the proposed work outperforms the QOSTBC using maximum likelihood (which is the optimal decoder for 16-PSK) by about 2 dB and outperforms the channel estimated scenario by more than 4 dB. Although this robust result seems to show a great advantage of using the proposed approach, we should be careful as it is not quite fair to compare 16-QAM and 16-PSK formats under the proposed nonlinear scenario.

**Figure 22:**
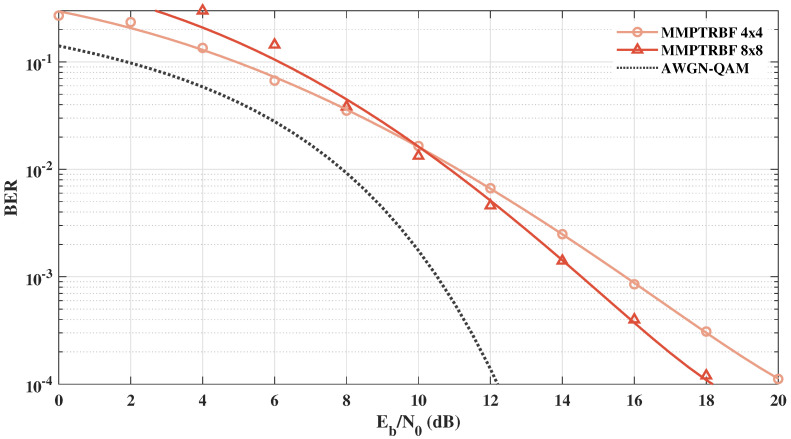
MMPTRBF with $M_{T}=M_{R}=4$ and $M_{T}=M_{R}=8$ antennas operating at the same bitrate with 16-QAM modulation.

Figure 22 shows the BER vs. $E_{b}/N_{0}$ results of the MIMO-PTRBF systems operating with $M_{T}=M_{R}=4$ and $M_{T}=M_{R}=8$ for 16-QAM modulation. Figure 22 highlights the increase in the diversity gain as a result of the increase in the number of transmitting antennas. Although the MMPTRBF with $M_{T}=M_{R}=8$ has the worst results for low values of $E_{b}/N_{0}$, for values above $E_{b}/N_{0}=10$ dB, the performance with $M_{T}=M_{R}=8$ surpasses the $M_{T}=M_{R}=4$ results by about 1.6 dB for BER $=10^{-3}$.

**Figure 23:**
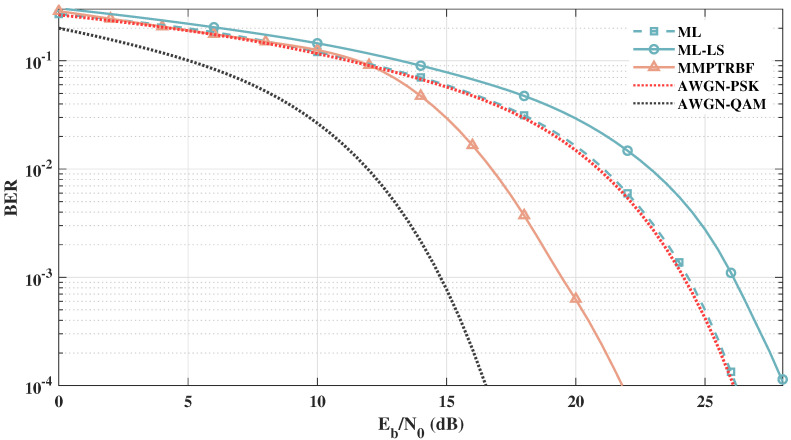
Systems with $M_{T}=M_{R}=4$ antennas operating at the same bitrate with 64-QAM (MMPTRBF) and 64-PSK (ML-QOSTBC and ML-LS-QOSTBC).

Figure 23 shows the BER vs. $E_{b}/N_{0}$ results of the MIMO-PTRBF with 64-QAM modulation to further investigate the proposed work for higher-order modulations with $M_{T}=M_{R}=4$ antennas. Figure 23 shows the potential of the proposed approach of working with high-order modulation and highlights the gains over the QOSTBC with 64-PSK and perfect channel estimation, by about 5 dB, and for the channel estimated scenario by about 7 dB. It is important to emphasize, once again, that it is not quite fair to compare 64-QAM and 64-PSK formats under the proposed nonlinear scenario.

**Figure 24:**
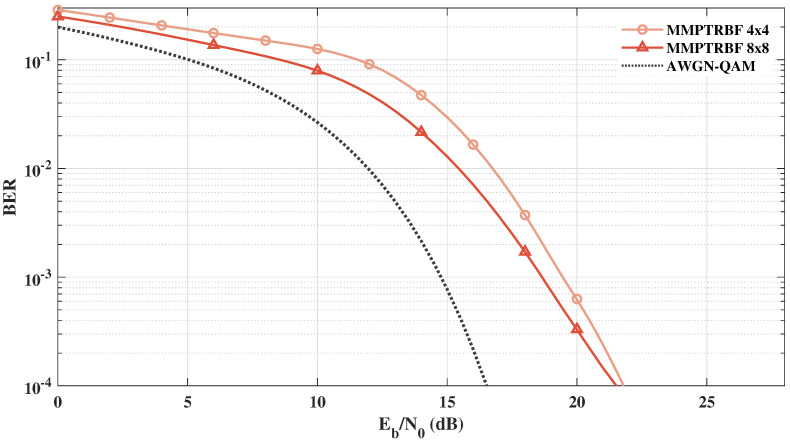
MMPTRBF with $M_{T}=M_{R}=4$ and 8 antennas operating at the same bitrate with 64-QAM modulation.

Figure 24 shows the BER vs. $E_{b}/N_{0}$ results of the MIMO-PTRBF systems operating with $M_{T}=M_{R}=4$ and $M_{T}=M_{R}=8$ for 64-QAM modulation. Figure 24 highlights the increase in the diversity gain as a result of the increase in transmitting antennas. When doubling the number of antennas from $M_{T}=M_{R}=4$ to $M_{T}=M_{R}=8$, the gain is about 0.87 dB for BER $=10^{-3}$.

## 5. Computational Complexities

Table 2 presents the computational complexities of the OSTBC and EOSTBC with ML decoding, both with the additional complexity of channel estimation, and the proposed scheme with MIMO-PTRBF for training and decoding operation modes. $M_{T}$ and $M_{R}$ are the number of transmitting and receiving antennas, $M_{s}$ is the number of transmitted symbols per MIMO block, *P* is the number of time samples per block of coded symbols, *A* is the constellation order (e.g., A=4 in the case of 4-QAM), and *N* is the number of neurons used in the PTRBF neural network. Since the exp(·) function can be easily implemented in hardware by lookup tables, multiplication is the most costly operation. One may note that, for $M_{T}\leq 8$, the complexity of the proposed algorithm is similar to the complexity of the OSTBC, for which no code exists for $M_{T}>8$ [54]. The case of QOSTBC is similar, for which no simplified ML metric exists for $M_{T}\neq 4$ [11,56].

Table 3 presents the computational complexities for the OSTBC, QOSTBC, and the proposed system, for $M_{T}=M_{R}=4$. In Table 3, N=100 neurons are used in the neural network, and maximum likelihood decoding is simulated with R=1. Note that generic maximum likelihood decoding refers to the minimization of Equation (5) for a rate one (R=1) coding scheme (e.g., it could decode the QOSTBC for the case $M_{T}=4$ at a higher computational cost), and it will be assumed as an upper bound for the computational complexities of the other quasi-orthogonal systems. Furthermore, appropriate modulation schemes are used to provide the desired transmission rate for the evaluated systems; i.e., 4-QAM for rate one code (R=1) and 16-QAM for half-rate code (R=1/2) [11].

Table 4 displays the computational complexities for $M_{T}=M_{R}=8$, when the PTRBF is equipped with N=150 neurons. QOSTBC is defined as not applicable since no simplified ML decoding metric has been presented in the literature. Thus, we need to rely on the usual ML metric to perform decoding, which implies the limitation of using QOSTBC combined with ML for a higher number of antennas in practical approaches.

Table 5 displays the computational complexities for $M_{T}=M_{R}=32$, when the PTRBF is equipped with N=600 neurons. As the OSTBC coding matrix is limited to eight antennas (see [54]), it is not applicable for $M_{T}=M_{R}=32$. As already mentioned, since there is no simplified ML metric to perform ML decoding with QOSTBC, it results in an explosion of computational complexity for $M_{T}=M_{R}=32$. On the other hand, the proposed approach can expand the number of antennas, maintaining a reasonable compromise between computational complexity and BER, as discussed in Section 4.

**Figure 25:**
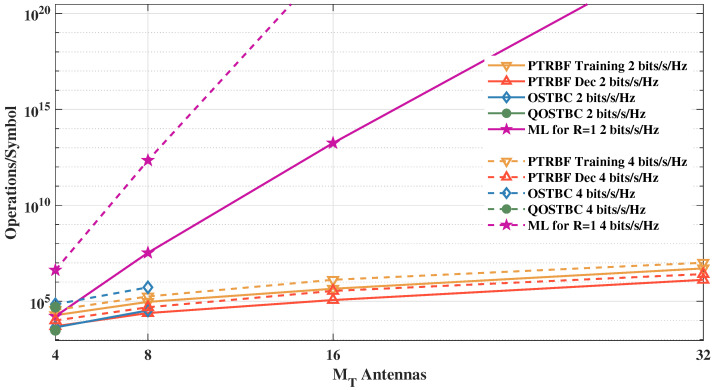
Computational complexities as a function of $M_{T}=M_{R}$.

The decoding computational complexities, shown in Figure 25, are addressed in terms of real-valued multiplications per MIMO symbol, as a function of $M_{T}=M_{R}$ antennas. The orthogonal and quasi-orthogonal systems are not illustrated for the entire simulation range in Figure 25 due to the absence of coding matrices and the simplification of the ML metric for configurations with $M_{T}=M_{R}>8$ [11].

## 6. Conclusions

This work proposes a novel MIMO scheme for M-QAM systems that aims to achieve diversity gain for any number of antennas and at a lower computational cost when compared with traditional methods. The presented architecture is based on existing systems but with substantial improvements in the coding and decoding methods, based on conventional MIMO-OFDM systems with quasi-orthogonal coding but implemented with complex-valued Radial Basis Functions neural networks. The state-of-the-art algorithms and the proposed approach have been simulated in MATLAB to measure their relative performance under fading scenarios.

Based on the synergistic combination of the coding and decoding algorithms presented in Section 2, the proposed MIMO-PTRBF system is discussed and analyzed in Section 3. The main functional features of the proposed architecture can be summarized as follows: (1) the proposed coding algorithm generalizes the generation of quasi-orthogonal coding matrices, (2) the MIMO-PTRBF algorithm decodes the signal with satisfactory performance and feasible computational cost, presenting low steady-state MSE with fast convergence, and (3) the proposed approach seems practically feasible, at least for 32 × 32 MIMO systems, which are simulated in this work. We conjecture the practical feasibility of higher-order systems if faster hardware, such as FPGAs, is used.

The MIMO-PTRBF algorithm has been proposed in this work to implement massive MIMO schemes as an alternative to the classic MIMO-OSTBC systems under maximum likelihood detection. Simulations have shown that the proposed technique has a great potential to improve the signal-to-noise ratio at the receiver, with competitive computational complexity. Although there are recent works in the literature proposing techniques for MIMO decoding, they are focused on reducing computational complexity at the cost of performance and are limited by the simulated ML decoding. In this work, results show that the proposed approach achieves better results than ML decoding for higher-order modulation schemes with nonlinearities from power amplifiers, keeping a competitive computational complexity. Moreover, the proposed system is easily scalable in terms of the number of antennas, meaning that a wide range of transmitting and receiving antennas can be used. This is especially important for the next generations of mobile communications, such as 5G, 6G, and probably beyond.

The proposed architectures and algorithms find potential applications in some configurations of the next generations of wireless systems. For example, some specialized hardware improvements currently aim exclusively at real-time neural network algorithms. These are intended to be implemented in low-power graphical processing units (LPGPUs), favoring the speed and energy consumption of these algorithms. Therefore, the proposed architecture will be able to work with low-power consumption devices, with the ability to handle the distortions of nonlinear power amplifiers while maintaining a fast convergence rate. It should be emphasized that a fast convergence characteristic is essential for wireless channels with dynamic fluctuations.

This paper addresses some crucial aspects of MIMO-OFDM coding and decoding schemes for quasi-static channels. A complementary analysis of dynamic scenarios is also presented. We conjecture that the proposed work may be further improved using additional techniques, such as a mathematical approach for designing an optimum adaptive configuration.

Furthermore, it would be interesting to study and validate the proposed architecture for dynamic scenarios. In addition, as challenging and promising future work, the proposed algorithm can be adapted and implemented in advanced optical communication systems with Spatial Division Multiplexing (SDM), which is similar to a MIMO wireless system.

## 7. Patents

A patent application with the results presented in this paper is being prepared by Inova—UNICAMP Innovation Agency.

## Figures and Tables

**Table 1:** Table 7.7.2-2. TDL-B.

Tap #	Normalized Delay	Power (dB)	Tap #	Normalized Delay	Power (dB)
1	0.0000	0.00	13	1.1021	−4.80
2	0.1072	−2.20	14	1.2756	−5.70
3	0.2155	−4.00	15	1.5474	−7.50
4	0.2095	−3.20	16	1.7842	−1.90
5	0.2870	−9.80	17	2.0169	−7.60
6	0.2986	−1.20	18	2.8294	−12.2
7	0.3752	−3.40	19	3.0219	−9.80
8	0.5055	−5.20	20	3.6187	−11.4
9	0.3681	−7.60	21	4.1067	−14.9
10	0.3697	−3.00	22	4.2790	−9.20
11	0.5700	−8.90	23	4.7834	−11.3
12	0.5283	−9.00			

**Table 2:** Computational complexities.

Decoder	Multiplications	Additions	exp(·)
OSTBC ${}^{†}$	$\left(2PM_{R}+4\right)M_{s}A+ch$	$\left(3PM_{R}+3\right)M_{s}A+ch$	ch
QOSTBC ${}^{†}$	$24M_{R}\left(M_{s}/2\right)A^{2}+ch$	$16\left(M_{R}-1\right)\left(M_{s}/2\right)A^{2}+ch$	ch
ML${}^{*}$ with R=1	$M_{R}M_{T}P\left(A^{M_{s}}\right)+ch$	$M_{R}M_{T}\left(P-1\right)\left(A^{M_{s}}\right)+ch$	ch
MIMO-PTRBF (train)	$12NM_{S}+12N+8NM_{R}P+2M_{s}$	$6NM_{R}P+12NM_{s}-2N$	2N
MIMO-PTRBF (decoding)	$2NM_{R}P+2N+4NM_{s}$	$4NM_{R}P+4NM_{s}-2N-2M_{s}$	2N

${}^{†}\;ch$ refers to the additional complexity of channel estimation.

**Table 3:** Computational complexities for $M_{T}=M_{R}=4$ and 2 bits/s/Hz.

Decoder	Multiplications	Additions	exp(·)
OSTBC ${}^{†}$	$4.35\times 10^{3}+ch$	$6.34\times 10^{3}+ch$	ch
QOSTBC ${}^{†}$	$3.07\times 10^{3}+ch$	$1.54\times 10^{3}+ch$	ch
ML for R = 1 ${}^{†}$	$1.64\times 10^{4}+ch$	$1.23\times 10^{4}+ch$	ch
MIMO-PTRBF (train)	$1.88\times 10^{4}$	$1.46\times 10^{4}$	200
MIMO-PTRBF (test)	$5.00\times 10^{3}$	$7.79\times 10^{3}$	200

${}^{†}\;ch$ refers to the additional complexity of channel estimation.

**Table 4:** Computational complexities for $M_{T}=M_{R}=8$ and 2 bits/s/Hz.

Decoder	Multiplications	Additions	exp(·)
OSTBC ${}^{†}$	$3.33\times 10^{4}+ch$	$4.95\times 10^{4}+ch$	ch
QOSTBC ${}^{†}$	Not applicable	Not applicable	Not applicable
ML for R = 1 ${}^{†}$	$3.36\times 10^{7}+ch$	$2.94\times 10^{7}+ch$	ch
MIMO-PTRBF (train)	$9.30\times 10^{4}$	$7.23\times 10^{4}$	300
MIMO-PTRBF (test)	$2.43\times 10^{4}$	$4.29\times 10^{4}$	300

${}^{†}\;ch$ refers to the additional complexity of channel estimation.

**Table 5:** Computational complexities for $M_{T}=M_{R}=32$ and 2 bits/s/Hz.

Decoder	Multiplications	Additions	exp(·)
OSTBC ${}^{†}$	Not applicable	Not applicable	Not applicable
QOSTBC ${}^{†}$	Not applicable	Not applicable	Not applicable
ML for R = 1 ${}^{†}$	$6.04\times 10^{23}+ch$	$5.86\times 10^{23}+ch$	ch
MIMO-PTRBF (train)	$5.15\times 10^{6}$	$3.92\times 10^{6}$	1200
MIMO-PTRBF (test)	$1.31\times 10^{6}$	$2.53\times 10^{6}$	1200

${}^{†}\;ch$ refers to the additional complexity of channel estimation.

## Data Availability

Not applicable.

## Appendix A

**Definition** **A1.**
*A non-monotone and differentiable transformation $g:R^{N}\mapsto R^{N}$, composed of M-real solutions, yields a multivariate change of probability density function:*

$$f_{Y}\left(y\right)=\sum_{m=1}^{M}f_{X}\left(x\right)\left|J\left(g_{m}^{-1}\left(y\right)\right)\right|,$$

*in which $x,y\in R^{N}$ are random vectors, J(·) is the determinant of the Jacobi operator, and $g_{m}^{-1}$ is the inverse transformation of g, regarding the mth real solution (see [65], p. 322 and [66], pp. 69–70).*

**Theorem** **A1.**
*If $x\in R^{N}$ is an input random vector of a kernel function $g\left(x\right)=y≜\exp\left\{-\frac{||x-{\gamma ||}_{2}^{2}}{z}\right\}$, with constant $\gamma \in R^{N}$ and z∈R; then, g performs a trans-dimensional transformation of probability density function from $f_{X}\left(x\right)$ to $f_{Y}\left(y\right)$.*

**Proof.** Let the Gaussian kernel function $g\left(x,\gamma ,z\right)≜\exp\left\{-\frac{||x-{\gamma ||}_{2}^{2}}{z}\right\}$, with constant center vector γ and variance *z*, be denoted by y=g(x), since it is only dependent on the random vector $x\in R^{N}$. Furthermore, let $y≜{\left[g\left(x\right)\;y_{2}\;\cdots y_{N}\right]}^{T}$ be the auxiliary expanded vector of mapping. With Definition A1, we have the following transformation of the probability density function (PDF):

(A1) $$f_{Y}\left(y\right)=\sum_{m=1}^{M}f_{X}\left(x\right)\left|J\left(g_{m}^{-1}\left(y\right)\right)\right|.$$

However, we need to marginalize the extra dimensions of y in (A1) to obtain the PDF of the desired random variable $f_{Y}\left(y\right)$:

(A2) $$f_{Y}\left(y\right)=\sum_{m=1}^{M}\int f_{X}\left(x\right)\left|J\left(g_{m}^{-1}\left(y\right)\right)\right|dy_{2}\cdots dy_{N}.$$

Then, finding some non-monotone function $h\left(y\right)=h\left(g(x)\right)=f_{X}\left(x\right)$, in which h(y) is not a function of the expanded auxiliary terms $y_{2}$, $y_{3}$, ⋯, $y_{N}$, we can rewrite (A2) as

(A3) $$f_{Y}\left(y\right)=\sum_{m=1}^{M}h_{m}\left(y\right)\int \left|J\left(g_{m}^{-1}\left(y\right)\right)\right|dy_{2}\cdots dy_{N},$$

where the right-hand term under the integral is called integral Jacobian, and it is responsible for the volume correction [67] of the trans-dimensional transformation. In addition, as the Gaussian kernel is a non-monotone function with two real solutions (due to the Euclidean norm), (A3) results in

(A4) $$f_{Y}\left(y\right)=h_{1}\left(y\right)\int \left|J\left(g_{1}^{-1}\left(y\right)\right)\right|dy_{2}\cdots dy_{N}+h_{2}\left(y\right)\int \left|J\left(g_{2}^{-1}\left(y\right)\right)\right|dy_{2}\cdots dy_{N},$$

which is the trans-dimensional transformation of PDF performed by a Gaussian kernel function. □

**Corollary** **A1.**
*For any complex-valued vectors of same size $x,y\in C^{N}$ and a scalar z∈C, a kernel function $f\left(x,y,z\right)≜\exp\left\{-\frac{||ℜ\left(x\right)-ℜ\left(y\right){\left|\right|}_{2}^{2}}{ℜ\left(z\right)}\right\}+j\exp\left\{-\frac{||ℑ\left(x\right)-ℑ\left(y\right){\left|\right|}_{2}^{2}}{ℑ\left(z\right)}\right\}$ have real and imaginary boundaries between 0 and 1, if ℜ(z)>0 and ℑ(z)>0.*

**Proof.** The proof is straightforward using Theorem A1 of [35], where the real-valued analyses must be independently performed for the real and imaginary components of x, y, and z. □

**Corollary** **A2.**
*If $x\in C^{N}$ is a complex-valued input random vector of a complex-valued kernel function $g\left(x\right)=y≜\exp\left\{-\frac{||ℜ\left(x\right)-ℜ\left(\gamma \right){\left|\right|}_{2}^{2}}{ℜ\left(z\right)}\right\}+j\exp\left\{-\frac{||ℑ\left(x\right)-ℑ\left(\gamma \right){\left|\right|}_{2}^{2}}{ℑ\left(z\right)}\right\}$, with constant $\gamma \in C^{N}$ and z∈C, then g performs a complex-valued trans-dimensional transformation of probability density function from $f_{ℜ\left(X\right)}\left(ℜ\left(x\right)\right)+jf_{ℑ\left(X\right)}\left(ℑ\left(x\right)\right)$ to $f_{ℜ\left(Y\right)}\left(ℜ\left(y\right)\right)+jf_{ℑ\left(Y\right)}\left(ℑ\left(y\right)\right)$, with independent real and imaginary components.*

**Proof.** The proof is straightforward using Theorem A1, where the real-valued analyses must be independently performed for the real and imaginary components of x and *y*. □

**Theorem** **A2.**
*If $x\in C^{K}$ is a stationary complex-valued input random vector of a PT-RBF, then the matrix of synaptic weights $W\left[k\right]\in C^{M\times N}$ converges in the mean to the optimum matrix of synaptic weights $W_{o}$ when k→∞, if the matrix of center vectors and the vector of variances are constants.*

**Proof.** Let the PT-RBF output $y\left[k\right]=W\left[k\right]\phi \left[k\right]\in C^{M}$, where $\phi \left[k\right]\in C^{N}$ is the vector of the neuron output. Furthermore, let the update function of the matrix of synaptic weights $W\left[k+1\right]=W\left[k\right]+\eta_{w}e\left[k\right]\phi^{H}\left[k\right]\in C^{M\times N}$, where $\eta_{w}$ is the adaptive step and $e\left[k\right]\in C^{M}$ is the vector of errors. We can assume without loss in generality that the desired response is

(A5) $$d\left[k\right]=W_{o}\phi \left[k\right]+q\left[k\right]\in C^{M},$$

where $q\left[k\right]\in C^{M}$ is a complex-valued white Gaussian noise vector, with zero mean and variance $\sigma_{q}^{2}$, which is uncorrelated with ϕ[k]. Then, substituting (A5) into the error equation e[k]=d[k]−y[k], we have

(A6) $$e\left[k\right]=W_{o}\phi \left[k\right]+q\left[k\right]-W\left[k\right]\phi \left[k\right].$$

Thus, replacing (A6) into the update function of the matrix of synaptic weights:

(A7) $$W\left[k+1\right]=W\left[k\right]+\eta_{w}W_{o}\phi \left[k\right]\phi^{H}\left[k\right]+\eta_{w}q\left[k\right]\phi^{H}\left[k\right]-\eta_{w}W\left[k\right]\phi \left[k\right]\phi^{H}\left[k\right].$$

Subtracting $W_{o}$ from both sides of (A7), the synaptic weights error matrix $V\left[k\right]=W\left[k\right]-W_{o}$ can be given by

(A8) $$V\left[k+1\right]=V\left[k\right]-\eta_{w}V\left[k\right]\phi \left[k\right]\phi^{H}\left[k\right]+\eta_{w}q\left[k\right]\phi^{H}\left[k\right].$$

Applying the expectation operator to both sides of (A8):

(A9) $$E\left(V[k+1]\right)=E\left(V\left[k\right]-\eta_{w}V\left[k\right]\phi \left[k\right]\phi^{H}\left[k\right]+\eta_{w}q\left[k\right]\phi^{H}\left[k\right]\right),$$

and employing the independence assumptions of W[k]⊥⊥ϕ[k] and q[k]⊥⊥ϕ[k]:

(A10) $$E\left(V[k+1]\right)=E\left(V[k]\right)-\eta_{w}E\left(V[k]\right)E\left(\phi \left[k\right]\phi^{H}\left[k\right]\right)+\eta_{w}E\left(q[k]\right)E\left(\phi^{H}\left[k\right]\right),$$

which results in:

(A11) $$E\left(V[k+1]\right)=E\left(V[k]\right)\left[I-\eta_{w}E\left(\phi \left[k\right]\phi^{H}\left[k\right]\right)\right]=E\left(V[k]\right)\left[I-\eta_{w}R_{\phi \phi }\right],$$

where I is the identity matrix and $R_{\phi \phi }$ is the correlation matrix of the neuron outputs. As $R_{\phi \phi }$ is Hermitian and positive semidefinite (see [68], pp. 387, 469), it can be rotated into a diagonal matrix by the unitary transformation $R_{\phi \phi }=Q\Lambda Q^{H}$, in which $\Lambda =\mathrm{diag}\left(\lambda_{1}\;\lambda_{2}\;\cdots \lambda_{N}\right)$ is the diagonal matrix of real and positive eigenvalues of $R_{\phi \phi }$ (see [68], p. 471), in the form of $\lambda_{1}\geq \lambda_{2}\geq \cdots \geq \lambda_{N}$, and $Q\in C^{N\times N}$ is the orthonormal matrix of eigenvectors that diagonalizes $R_{\phi \phi }$ through a similarity transformation [69]. Then, rotating $E\left(V[k]\right)$ by the matrix of eigenvectors Q—i.e., $\bar{V}\left[k\right]=E\left(V[k]\right)Q$—decouples the evolution of its coefficients. By means of this rotation, we can express the modes of convergence (see [70], p. 77) of (A11) as

(A12) $$\bar{V}\left[k+1\right]=\bar{V}\left[k\right]\left(I-\eta_{w}\Lambda \right).$$

As $\left(I-\eta_{w}\Lambda \right)$ is diagonal and the *n*th row of $\bar{V}\left[k\right]$ represents the projection of $E\left(V[k]\right)$ onto the *n*th eigenvector of $R_{\phi \phi }$, all elements of $\bar{V}\left[k\right]$ evolve independently. Hence, (A12) converges to zero if $\left|1-\eta_{w}\lambda_{n}\right|<1$ (see [69], p. 84). As the fastest mode of convergence corresponds to the maximum eigenvalue $\lambda_{max}$, using the identity $\lambda_{1}=\lambda_{max}\leq \mathrm{tr}\left(R_{\phi \phi }\right)$ (see [70], p. 77), the condition for the convergence in the mean becomes

(A13) $$0<\eta_{w}<\frac{2}{\lambda_{max}}\approx \frac{2}{\mathrm{tr}(R_{\phi \phi })}.$$

As the trace of $R_{\phi \phi }$ is equal to the product of the number of neurons outputs and the respective signal power, the adaptive step bound is given by

(A14) $$0<\eta_{w}<\frac{2}{NE\left({\left|\phi [k]\right|}^{2}\right)}.$$

Note that the convergence in the mean of both PT-RBF and complex LMS is similar (see [70], p. 77), and it is natural in some way, considering that after the trans-dimensional transformation step of the PT-RBF, both algorithms have comparable architectures. However, in view of Corollary A2, $E({\left|\phi [k]\right|}^{2})$ can be difficult to obtain. To circumvent this issue, using Corollary A1, we can replace ϕ[k] by its maximum value (1+j) into (A14), which yields

(A15) $$0<\eta_{w}<\frac{2}{N{\left|1+j\right|}^{2}}=\frac{1}{N},$$

which is the adaptive step bound for the convergence in the mean. □
